# Rationale and methods of the iFightDepression study: A double-blind, randomized controlled trial evaluating the efficacy of an internet-based self-management tool for moderate to mild depression

**DOI:** 10.1186/s12888-017-1306-2

**Published:** 2017-04-19

**Authors:** Azucena Justicia, Matilde Elices, Ana Isabel Cebria, Diego J. Palao, Jesús Gorosabel, Dolors Puigdemont, Javier de Diego-Adeliño, Andrea Gabilondo, Alvaro Iruin, Ulrich Hegerl, Víctor Pérez

**Affiliations:** 1 Centro de Investigación Biomédica en Red de Salud Mental (CIBERSAM), Madrid, Spain; 2 Departament de Psiquiatria i Medicina Legal, Universitat Autònoma de Barcelona, Barcelona, Cerdanyola del Vallès Spain; 3 Institut de Neuropsiquiatria i Addiccions (INAD), PSMar, Barcelona, Spain; 4 Institut Hospital del Mar d’Investigacions Mèdiques (IMIM), Barcelona, Spain; 5 Parc Taulí Hospital Universitari, Sabadell, Spain; 6 Centro de Salud Lagasca, Madrid, Spain; 7 Departament de Psiquiatria, Hospital de la Santa Creu i Sant Pau, Institut d’Investigació Biomèdica Sant Pau, Barcelona, Spain; 8 Outpatient Mental Health Care Network, Osakidetza- Basque Health System, Biodonosti Health Research Institute, San Sebastian, Spain; 9 University Hospital, Department of Psychiatry and Psychotherapy, University of Leipzig, Leipzig, Germany; 10 German Depression Foundation, Depression Research Centre, Leipzig, Germany

**Keywords:** Depression, Cognitive behavioural therapy, Internet-based, Self-management, Primary care, Randomized controlled trial

## Abstract

**Background:**

During the last decade online interventions have emerged as a promising approach for patients with mild/moderate depressive symptoms, reaching at large populations and representing cost-effective alternatives. The main objective of this double-blind, randomized controlled trial is to examine the efficacy of an internet-based self-management tool (iFightDepression) for mild to moderate depression as an add-on to treatment as usual (TAU) versus internet-based psychoeducation plus TAU.

**Methods:**

A total of 310 participants with major depression disorder (MDD) will be recruited at four different mental-health facilities in Spain. Participants will be randomly allocated to one of two study arms: iFightDepression (iFD) tool + TAU vs. internet-based psychoeducation + TAU. Both interventions last for 8 weeks and there is a 12 weeks follow up. The primary outcome measure is changes in depressive symptoms assessed with the Hamilton Depression Rating Scale. Additionally, pre-post interventions assessments will include socio-demographic data, a brief medical and clinical history and self-reported measures of depressive symptoms, quality of life, functional impairments and satisfaction with the iFD tool.

**Discussion:**

iFightDepression is an easy-prescribed tool that could increase the efficacy of conventional treatment and potentially reach untreated patients, shortening waiting lists to receive psychological treatment. Confirming the efficacy of the iFD internet-based self-management tool as an add-on treatment for individuals with mild to moderate depression will be clinically-relevant.

**Trial registration:**

Registration number NCT02312583. Clinicaltrials.gov. December 4, 2014.

## Background

According to the World Health Organization (WHO) predictions, by 2030 depression will be the leading cause of disease burden globally. Depression is not only associated with disability and with enormous individual impairments, but also it entails high economic cost for society [1, 2].

Depression is a recurrent disorder, meaning that one individual will suffer an average of four depressive episodes during his/her life [3]. Establishing an effective treatment at the early stages of the disease is fundamental for the successful prognosis of the disorder [4]. Although several treatment options have been developed for depression, a large proportion of individuals do not have access to these treatments and some of them remain untreated [5]. Furthermore, contrary to what is suggested in international guidelines for the management of mild and moderate depression symptoms, the majority of General Practitioners (GPs) tend to prescribe antidepressant medications [6, 7].

In this context, online interventions appear as promising treatment approaches that can reach a large majority of patients [8, 9], while at the same time, representing cost-effective alternatives [10]. For clients, internet-based programs offer some advantages. These include: anonymity, avoiding the stigma associated with seeing a therapist and the option to do the treatment at any time and place, at the client’s own pace, for example, at home [11, 12]. Therapist involvement depends on the intervention, but in general, most programs aim at decreasing the therapist workload. Most internet-based treatments are framed in cognitive behavioral therapy (CBT) and are developed to treat individuals with mild to moderate depressive symptoms that are usually visited in primary care settings. In a meta-analysis of randomized-controlled trials comparing the effects of guided self-help and face-to-face psychotherapies, it was concluded that both interventions have comparable effects. Thus, recommending the implementation of guided self-help interventions in routine care [13].

In the context of the European project Preventing Depression and Improving Awareness through Networking in the European Union (PREDI-NU) [14], the European Alliance Against Depression (EAAD) created the iFightDepression (iFD) tool. The EAAD is a consortium of different countries around the world that use an evidence-based approach for improving care of depressed persons and prevent suicidal behavior [15]. IFD is an internet-based self-management tool for moderate to mild depression designed as a complementary intervention to standard care. IFD is based on cognitive behavioral therapy and structured in seven different modules.

### Study objectives

The aim of this double blind, randomized controlled trial (RCT) is to examine the efficacy of the iFightDepression as an add-on to treatment as usual (TAU) for patients with mild to moderate depressive symptoms versus internet-based psychoeducation plus TAU. The primary outcome measure will be changes in depressive symptoms as measured with the Hamilton Depression Rating Scale [16]. Secondly, we aim at examining the effects of this intervention on other variables including quality of life and functioning.

## Methods

### Design

We designed an 8-week, double-blind, randomized controlled trial, with two treatment arms: iFightDepression tool + TAU and internet-based psychoeducation + TAU. Participants will be randomly allocated (1:1) after the first assessment visit. Randomization will be performed electronically by an independent study researcher. Participants in both treatment arms will be followed via internet during a 12-week period.

### Study participants

A total of 310 participants with major depressive disorder (MDD) diagnosis will be included in the study. Diagnosis will be made according DSM-IV-TR criteria and participants will be recruited from different mental health services (i.e., Red de Salud Mental de Guipúzkoa, Hospital del Mar, Hospital de la Santa Creu i Sant Pau, Hospital Parc Taulí and Hospital Universitario de la Princesa) and primary care facilities in their catchment areas. Sample size was established in order to detect an effect size of d = 0.2 with 95% power (SE = 0.5) and assuming a conservative correlation coefficient between pre-post measures of 0.5 and a drop-out of 20%. Sample size was calculated using G*Power 3.1.7 software.

Individuals meeting the following criteria will be eligible: 1) Participants of both genders 18 years old and older, 2) MDD diagnosis according to DSM-IV-TR criteria with mild-moderate symptoms according to the CGI-severity (scores ≤4), 3) minimum knowledge of internet use and availability of an internet device (tablet, computer, smartphone) and 3) given written informed consent. Exclusion criteria are: 1) suicidal ideation assessed by the HDRS and/or clinical interview, 2) psychotic symptoms related or not related to a mood disorder, 3) severe comorbidity axis I and axis II diagnosis, 4) currently receiving standard face-to-face CBT.

### Procedure

**Fig. 1:**
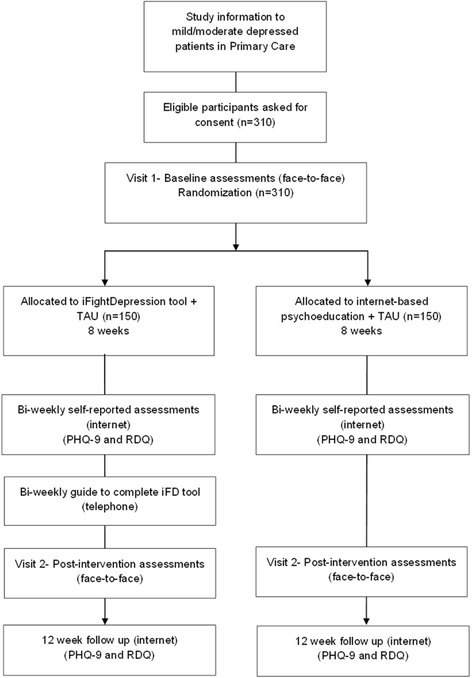
Flowchart of participants

Eligible participants will be identified through the different sites by a case manager, mainly, by the participant’s general practitioner. However, other professionals (i.e., psychiatrist, psychologist or community mental health nurses) with experience in the assessment and diagnosis of depression would be also able to refer participants to the study. These professionals will verify inclusion/exclusion criteria and will inform patients about the study. Participants who confirm their interest in participating, will be contacted by phone within 1 week to schedule the first assessment visit (face-to-face). Each site will designate a highly trained psychologist who will conduct the first face-to-face visit in order to confirm that participants met inclusion/exclusion criteria and to obtain the written informed consent. Participant’s assessment will be recorded in the electronic Case Report Form (e-CRF). In this assessment visit, and regardless of the treatment arm the patient will be assigned to, the second face-to-face assessment visit (week 8) will be booked. Randomization to one of the two conditions (i.e., iFightDepression tool + TAU or internet-based psychoeducation + TAU) will be electronically performed by the e-CRF once the patient data is introduced. The researcher who conducts assessment visits will remain blind to participant’s allocation. After the first visit, participants in both arms will received an email with instructions to be followed. The intervention group will receive instructions to create an account and to work within the iFD tool. In addition, the intervention group will receive a telephone call to make sure that participants activated successfully their user accounts. Then, they will be contacted bi-weekly by a mental health professional (specialized nurse or psychologist), to work through the material and to provide technical support). During the 8 weeks of the trail all participants will be follow-up by e-mail in order to complete the PHQ-9 and RDQ. Participants will have to complete the PHQ-9 and the RDQ again at week 12. The study protocol was developed in accordance with the SPIRIT (Standard Protocol Items: Recommendations for Interventional Trials) guidelines [17]. A flowchart of participants is shown in Fig. 1.

### Ethical issues

All participants will be provided with an overview of the study’s aims and characteristics summarized in the Participants Information Sheet (PIS). The voluntarily character of the study will be specified, indicating that withdrawal from the study is permitted at any time, without interfering with the usual treatment. Written informed consent will be obtained from all participants before proceeding with randomization. The study will be performed according to the ethical standards stated in the Declaration of Helsinki and its subsequent updates. The Ethics Committee Board of the Parc de Salut Mar approved the study protocol in October 2014. Data registered in the e-CRF will be coded as to ensure the confidentiality of participant’s identity. The access to the code key will be restricted to the principal investigator of the study, and will be stored separately in a safe place.

### Description of the interventions

#### Intervention group (iFightDepression tool + TAU)

IFightDepression (https://tool.ifightdepression.com) is an internet-based self-management tool designed as a complementary intervention to standard care for patients with mild/moderate symptoms of depression. IFD was created by the European Alliance against Depression (EAAD) and is based in cognitive behavioral therapy (CBT). The IDF tool is structured in seven core modules (workshops) relating to behavioral activation (monitoring and planning daily activities), cognitive restructuring (identifying and changing unhelpful thoughts), sleep regulation, mood monitoring, and healthy lifestyle habits. Each module consists of written information, associated tasks and corresponding worksheets to consolidate learning and promote self-monitoring. A guide on how to complete the worksheets is provided in each module. Worksheets can be printed or completed on-line and are saved in the participants profile for latter review. Patients are encouraged to work with the worksheets for an entire week so the whole content of the program is developed to be completed in 8 weeks. An outline of each module can be found in Table 1. In addition, the tool has a “Frequently asked questions (FAQ)” section and a section “About IDF”, in which participants can find information regarding the causes of depression, the use of the tool, and further reading on cognitive behavioral therapy.

**Table 1:** Content outline for iFightDepression tool-intervention group

Module	Content/Aim	Worksheet
1 thinking, feeling and doing	Recognizing the relationship between thoughts, feelings and behavior.	Activity Monitoring
2 Sleep depression	Gaining awareness of the influence of sleep on depression.	Sleep Diary
3 Planning and doing enjoyable things	Building up positive activities in the daily life.	Planning Ahead
4 Getting things done	Developing skills for problem solving and to get things done.	Getting things done
5 Identifying negative thoughts	Understanding the link between thoughts and mood, and learning how to recognize cognitive distortions.	Events, Thoughts and Reactions
6 Changing negative thoughts	Learning how to challenge negative thoughts and how to replace them with more positive alternatives.	Thought Challenge
7 Feel better all around: Healthy Lifestyle	Giving tips for developing a healthy lifestyle in general. Including healthy diet, exercise and sleep.	Sleep Worry Planner

For the purpose of this study and to facilitate the recruitment, the IFD tool is available in three languages: Spanish, Catalan and Basque, and patients are allowed to choose among versions according to their language preferences. Participants can access the PHQ-9 at any time, to assess their depression symptoms. A guide for interpreting the PHQ-9 scores is provided. If the patient responds positively to the item regarding suicide (i.e., “Thoughts that you would be better off dead or of hurting yourself in some way”) a message recommending seeking for emergency care will appear on the top of the screen. In addition, emergency contacts for Spain are listed on the website. The use of IDF tool is complementary to TAU which consists of monthly visits with the GP and pharmacological treatment with an ISRSS if needed.

#### Active control group (internet-based psychoeducation + TAU)

Internet-based psychoeducation consists of a webpage containing information about MDD and healthy habits. This webpage (www.ifightdepression.com) was also designed by the EAAD consortium, and unlike the tool, workshops and tasks are lacking. The psychoeducative content of this website is restricted to information regarding healthy habits (healthy eating, physical activity, positive social contact and daily organization). In addition, information on the following topics is included: what is depression, signs and symptoms, diagnosing depression, subtypes of depression, brain function in depression, causes and an overview of depressions treatment (both pharmacological and psychological). Participants on this condition can also complete the online PHQ-9. This is an open-access webpage (i.e., a username and password are not required). Participants allocated to this condition will receive an e-mail with the page link and will be instructed to navigate freely throughout their content. As in the intervention group, TAU consists of monthly visits to the GP together with pharmacological treatment (if needed).

### Study measures

Different types of variables will be collected (see Table 2) including socio-demographic characteristics of the sample (i.e., gender, age, ethnic group, marital status, living arrangements, educational level, employment status and annual income) and an exhaustive clinical history [i.e., age at MDD onset, number of previous MDD episodes, days in inpatient hospitalization (if applied), axis I and axis II diagnosis, and current pharmacological treatment].

**Table 2:** Measures used by study period

Measure	Baseline assessments (Face to face)	Week 2 (Online)	Week 4 (Online)	Week 6 (Online)	Post-intervention assessment (Face to face)	12 weeks FU (online)
Demographic information (gender, age, marital status, etc.)	x					
Medical/Psychiatric History	x				x	
MINI (depression and suicide risk sections)	x				x	
Primary outcome measure:						
-HDRS	x				x	
Secondary outcome measure:						
-PHQ-9	x	x	x	x	x	x
-CGI-S					x	
-RDQ	x	x	x	x	x	x
-EuroQol-5D	x				x	
-FAST	x				x	
-Ad hoc satisfaction Questionnaire					x	

*Note*: *MINI* Mine-international Neuropsychiatric Interview, *HDRS* Hamilton Depression Rating Scale, *PHQ-9* Patient health Questionnaire, *CGI-S* Clinical Global Impression-Severity, *CGI-I* Clinical Global Impression-Improvement, *RDQ* Remission from Depression Questionnaire, *FAST* Functioning Assessment Short Test

### Diagnostic variables

Depression symptoms and suicide risk will be assessed using the specific sections of the Mini-International Neuropsychiatric Interview (MINI) [18], a short diagnostic structured interview. In addition, an ad hoc questionnaire was created for accounting for sociodemographic and clinical data.

### Primary outcome measure

The Hamilton Depression Rating Scale (HDRS) [16] will be the primary outcome measure. This is a clinician-administered scale. Each item on the questionnaire is scored on a 3- or 5-point scale, depending on the item.

### Secondary outcome measures

The following instruments will be used to assess other outcomes:

Patient Health Questionnaire (PHQ-9) [19] is an instrument for screening severity of depression according to DSM-IV-TR criteria. The tool rates the frequency of the symptoms during the last 2 weeks using a scale from 0 (not at all) to 3 (nearly every day).

Clinical Global Impression Severity and Improvement (CGI-S and CGI-I; [20]. The CGI-S scale would be used to evaluate the clinical severity of the participant in a scale from 1 to 7 (“normal, not ill at all” to “extremely ill”). Both a patient-rated version and a clinician-rated version will be used. The CGI-Improvement (CGI-I) will be also used, in order to evaluate the improvement between the baseline visit and the post-intervention assessment (from 1:“very much improved” to 7:“very much worse”).

Remission from Depression Questionnaire (RDQ; [21] is a self-reported instrument developed from a patient-centered perspective, including the aspects that they considered as part of depression remission. The RDQ evaluates depressive symptoms, positive mental health and satisfaction with life, well-being and social functioning.

EuroQol-5D questionnaire (EQ-5D-5 [22] is a widely used measure of health-related quality of life. The instrument is composed of two parts. Part one provides a self-reported description of health problems classified into five dimensions and patients have to rate the severity corresponding to each dimension in a 5-point scale. Part two consists of a 10 cm line Visual Analogue Scale (VAS) corresponding to the current state of subject’s health. The lowest extreme of this scale (0) corresponds to the worst imaginable health state, while the highest extreme (100) refers to the best imaginable health state.

Functioning Assessment Short Test FAST [23] is a brief instrument developed to assess the main functioning problems experienced by patients. It comprises 24 items assessing impairment or disability in six areas of functioning: autonomy, occupational functioning, cognitive functioning, financial issues, interpersonal relationships and leisure time. Each item is rated on a 4-point scale (0 = no difficulty, 1 = mild difficulty, 2 = moderate difficulty and 3 = severe difficulty).

An ad hoc satisfaction questionnaire was created for participants allocated to the intervention group.

### Statistical analysis plan

SPSS v22.0 will be used for statistical analyses. Primary analyses will be conducted on the Intention-To-Treat (ITT) sample. Missing data will be inspected and handled via full information maximum likelihood (FIML) or multiple imputations (MI) as appropriate.

The primary outcome is change in HDRS scores from baseline to post-treatment (8 weeks). For primary and secondary outcome measures, repeated measures ANOVAs will be computed to examine pre-post treatment differences between groups.

## Discussion

The present study aims at evaluating the efficacy of an internet-based self-management tool for moderate to mild depression compared with internet-based psychoeducation. Confirming the efficacy of the iFightDepression tool will entail important implications. As IFD could be easily prescribed it could potentially reach patients that usually remain untreated or have to wait for long-periods to receive psychological treatment. In addition, the availability of the both interventions in three different languages (Spanish, Catalan and Basque) aims at facilitating participant’s adherence. A strength of our study is that the recruitment will mainly take place in general practices, were patients with mild to moderate depression symptoms are often treated.

The risk of dropout constitutes one of the potential major obstacles of our study. We’ve tried to mitigate this by expanding our recruitment area to public health-care facilities located in three large areas of Spain (Catalonia, Madrid and Basque Country) and by providing specific language versions of the iFD tool and the psychoeducation web-page. As commented before, the design of our study privileges a naturalistic approach and therefore, participants will be recruited from primary care facilities in which most patients with mild/moderate symptoms of depression are visited and participants with concomitant ant depressive medication will be allowed in the study. Considering that the effects of the concomitant medication may impact our findings, a sub-analysis evaluating the potential effects of antidepressant will be conducted and participants will be excluded from the study if drastic changes in their medication took place during the study period.

In summary, the present study aims to provide evidence in regards to iFD tool as an add-on approach for individuals presenting mild to moderate symptoms of depression. Counting on a tool like iFD will facilitate its use in the public health care system, representing a treatment approach that can potentially reach a large amount of patients avoiding long waiting lists.

## Abbreviations

CBT: Cognitive behavioural therapy
CGI – I: Clinical global impression - improvement
CGI-S: Clinical global impression - severity
EAAD: European alliance against depression
e-CRF: Electronic clinical report form
FAST: Functioning assessment short test
HDRS: Hamilton depression rating scale
iFD: iFightDepression
ITT: Intention to treat
MDD: Major depressive disorder
MINI: Mini-international neuropsychiatric interview
PHQ-9: Patient health questionnaire
PIS: Patient information sheet
PREDI-NU: Preventing Depression and Improving Awareness through Networking in the European Union (PREDI-NU)
RDQ: Remission from depression questionnaire
TAU: Treatment as usual
WHO: World Health Organization
